# Development of Fully Flexible Tactile Pressure Sensor with Bilayer Interlaced Bumps for Robotic Grasping Applications

**DOI:** 10.3390/mi11080770

**Published:** 2020-08-12

**Authors:** Lingfeng Zhu, Yancheng Wang, Deqing Mei, Chengpeng Jiang

**Affiliations:** 1 State Key Laboratory of Fluid Power and Mechatronic Systems, School of Mechanical Engineering, Zhejiang University, Hangzhou 310027, China; lingfengzhu@zju.edu.cn (L.Z.);; 2 Key Laboratory of Advanced Manufacturing Technology of Zhejiang Province, School of Mechanical Engineering, Zhejiang University, Hangzhou 310027, China; 3 Research Center for Smart Sensing, Zhejiang Lab, Hangzhou 310000, China; chengpen@connect.hku.hk

**Keywords:** flexible tactile sensor, robotic hand, bilayer interlaced bumps, stretchable composites, grasping motions

## Abstract

Flexible tactile sensors have been utilized in intelligent robotics for human-machine interaction and healthcare monitoring. The relatively low flexibility, unbalanced sensitivity and sensing range of the tactile sensors are hindering the accurate tactile information perception during robotic hand grasping of different objects. This paper developed a fully flexible tactile pressure sensor, using the flexible graphene and silver composites as the sensing element and stretchable electrodes, respectively. As for the structural design of the tactile sensor, the proposed bilayer interlaced bumps can be used to convert external pressure into the stretching of graphene composites. The fabricated tactile sensor exhibits a high sensing performance, including relatively high sensitivity (up to 3.40% kPa^−1^), wide sensing range (200 kPa), good dynamic response, and considerable repeatability. Then, the tactile sensor has been integrated with the robotic hand finger, and the grasping results have indicated the capability of using the tactile sensor to detect the distributed pressure during grasping applications. The grasping motions, properties of the objects can be further analyzed through the acquired tactile information in time and spatial domains, demonstrating the potential applications of the tactile sensor in intelligent robotics and human-machine interfaces.

## 1. Introduction

Flexible and wearable electronics/devices have gained increasing interest due to their widespread potential applications in intelligent robotics [1,2,3], smart prosthetics [4,5], human-machine interface [6,7,8], and healthcare monitoring [9,10,11]. Tactile sensors, providing abundant firsthand tactile information perception like pressure [7], three-dimensional forces [12], slippage [13], friction [14], torque [15], and textures [16], have been paid particular attention as they have the essential capability to bridge the gaps between mankind, robots, and the external environment [17]. Among the diverse tactile parameters, pressure has played an essential role as many other parameters can be acquired from the tactile sensors through pressure detection. For example, a tactile sensor array for 3D force measurement of robotic hand [12] has been developed by combining a 2 × 2 pressure units into one 3D force unit. Normal and shear forces applied to the unit could be calculated by analyzing the differential relationship among the 2 × 2 pressure units. A tactile sensor for slippage and surface texture [13] has also been reported based on normal pressure detection. The acquired pressure information in the time domain was transformed into the frequency domain through discrete sequence wavelet transform, and the variations of the wavelet coefficient were utilized to detect the slippage and further recognize the surface texture. Thus, the pressure sensing capability can be the base for the robots to achieve the detection of diverse tactile information.

Poor flexibility and low sensitivity of conventional metal- and semiconductor-based tactile sensors have been hindering their applications towards the next-generation humanoid robots [18], which are far more similar to human beings from both appearance and functionality perspectives. Many studies have involved elastic polymers into the tactile sensors and successfully enhanced the flexibility, but the usage of metallic electrodes makes them not able to compliantly and conformably cover the irregular surfaces of the robotic hands [19,20]. The integration of the tactile sensors with robotic hands will generate significant influence on the sensing accuracy. Moreover, the flexibility does not only affect the integration between the sensors and the robots but also represents the compatibility of the sensors for different grasping motions as well as different grasping objects. For the humanoid robots, dexterous manipulation of objects is essential, which raises the requirements for the tactile sensors to precisely perceive the tactile information under different grasping motions towards objects with diverse shapes and properties. Therefore, further promotion in both flexibility and sensitivity of the tactile sensors is in urgent need.

Flexible tactile sensors have been developed based on various transduction mechanisms, such as capacitive [21], piezoresistive [22,23], piezoelectric [24], and triboelectric effects [25]. Among them, the piezoresistive type has exhibited intensive potentials due to their excellent flexibility, relatively high sensitivity, and broad measuring range [26,27]. Carbon nanotube (CNT) [22], graphene [27], and metal nanowires/nanoparticles [11,28] have been the most common choices to fabricate the piezoresistive tactile sensors, among which graphene is a considerable candidate owing to its superior electrical and mechanical properties [29]. For example, graphene films fabricated by chemical vapor deposition (CVD) and graphene foam from freeze-drying have been reported to work as the tactile sensing elements with ultrahigh sensitivity [30,31]. Whereas, the high-cost and complicated fabrication process limits their applications. Simpler methods like dip-coating have also been used to fabricate the graphene-based tactile sensors [10]. One common issue for the above methods is that these methods are difficult to fabricate customized distributed tactile sensor arrays that are compatible with the complicated and irregular surfaces of the humanoid robotic hands. Directly dispersing graphene into the polymers to obtain the conductive elastomers seems to be an efficient and economical way as the polymers have better manufacturability and flexibility to be shaped into the desired structures for robotic hands. But the uniformity of graphene dispersing in the polymers will be a crucial challenge.

Apart from the sensing materials, the structural design has also been of vital importance to the performance of the tactile sensors. Many studies have reported tactile sensors with micro- and nano-structures to greatly improve the pressure sensitivity, such as micropyramids [32], micropillars [33], nanorods [34], and nanocones [21]. Ultrahigh sensitivity has been achieved due to these superb structures, which is even capable of precisely detecting the weeny pulse beats from human arteries, but on the other hand, the sensing ranges of these sensors are usually small [30]. For example, a tactile sensor with CNT-graphene film and microstructured PDMS has been developed to possess a large sensitivity of 19.8 kPa^−1^, but in a really small range of only 0.3 kPa [35]. A tactile sensor with interlocked microdomes has also been fabricated to perform with a high sensitivity of 15.1 kPa^−1^ but possessed a very low range of 2 kPa [36]. Such tactile sensors with low sensing ranges cannot meet the demand of the robotic hands for grasping and manipulating various objects. Besides, to fabricate these sophisticated structures, micro-nano machining methods like microelectromechanical system (MEMS) technology was usually involved [32]. Such expensive and time-consuming procedures have also been a limitation. Hence, a novel structural design of the tactile sensor with an efficient fabrication process is necessary to balance the sensitivity and sensing range for robotic hand grasping.

Herein, we present a fully flexible tactile pressure sensor for distributed pressure sensing during robotic hand grasping. To achieve high flexibility and stretchability, graphene and silver nanoflakes were dispersed into the elastic silicone rubber to function as the pressure sensing cell and stretchable electrodes, respectively. A simple but efficient planetary mixing method was utilized to improve the dispersion uniformity of the nanofillers inside the polymeric matrix. A bilayer interlaced bump structure was designed for the tactile sensor to convert the external pressures of the sensor into the stretching of the graphene composites. The proposed structures could balance the trade-off between the sensitivity and sensing range and the FEM model has been established to verify the efficiency of the structures. Due to the prepared composites and structures, the tactile sensor exhibited a relatively high sensitivity of up to 3.40% kPa^−1^ in a wide range of 200 kPa. Further, the fabricated tactile sensor can be integrated with the humanoid robotic hand through the designed wearable buckles, and grasping experimental studies have been conducted for a hard cylinder object and a soft tennis ball. The distributed tactile information analyzed from time and spatial domains has shown possibilities to investigate the relationship between the tactile signals, robotic hand grasping motions, and even the properties of the grasped objects. The experimental results demonstrate the potential applications of the proposed tactile sensor in intelligent robots and human-machine interfaces.

## 2. Design of Flexible Tactile Pressure Sensor

### 2.1. Flexible Tactile Sensor with Bilayer Interlaced Bumps

**Figure 1:**
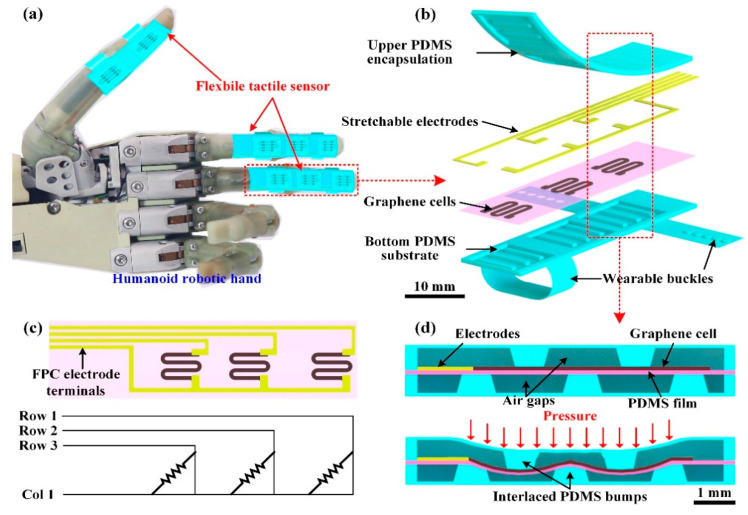
(**a**) Schematic view of the fully flexible tactile pressure sensor for the robotic hand; (**b**) The multilayer structure of the tactile sensor; (**c**) Electrode connection of the graphene cells; (**d**) The pressure-sensing mechanism of the tactile sensor.

Confronting these objectives to enhance the flexibility and sensitivity, a fully flexible tactile pressure sensor for the humanoid robotic hand has been proposed, and its schematic view is given in Figure 1. As shown in Figure 1a, the tactile sensor is comprised of three separate smaller sensors, which are mounted on the thumb, index finger, middle finger of the robotic hand, respectively. The sensors on the index and middle finger are identical with three sensing units, whereas, the sensor on the thumb possesses two units due to the structure and motion features of the robotic hand. The units in the sensor have the same structure and the multilayer structural design of the middle finger sensor is illustrated in Figure 1b. The sensor consists of four main layers: bottom substrate, graphene cells, stretchable electrodes, and upper encapsulation. The wearable buckles on the bottom substrate make it convenient for the tactile sensor to be worn on the robotic hand fingers.

The serpentine-patterned graphene cells are assigned on the polydimethylsiloxane (PDMS) film and function as the pressure-sensitive elements. As shown in Figure 1c, the stretchable electrodes are connected to the ends of the graphene cells for electrical transmission to measure the resistance of the graphene cells. In Figure 1c, the upper ends of the cells are connected with three individual row electrodes, while the lower ends are linked to one public column electrode. And the terminals of the stretchable electrodes are designed as the flexible printed circuit (FPC) to connect with peripheral measurement instruments. For a single unit in the sensor, its cross-sectional view in Figure 1d can describe its pressure sensing mechanism. The graphene cell on the PDMS film is sandwiched by the upper encapsulation and bottom substrate, in which there are two and three separate trapezoid bumps, respectively. The upper encapsulation and bottom substrate with the bumps are all composed of PDMS. The upper and lower bumps are distributed in the same spacing distance and together form the interlaced structure. As shown in Figure 1d, when a pressure is applied to the sensing unit, the pressure is converted to the graphene cell through the upper bumps. Owing to the gaps between the lower bumps under the graphene cell, the cell on the PDMS film is squeezed into the gaps and generate distinct tensile strains. Therefore, the pressure is converted to the stretching of the graphene cell through the interlaced bumps, further into the resistance change of the cell. The tactile sensor has been fabricated based on fully flexible and stretchable materials. The upper encapsulation and bottom substrate were made of PDMS through mold casting. The film beneath the graphene cells was prepared through PDMS coating. The graphene cells and stretchable electrodes have been fabricated by dispersing graphene nanoplates (GNP) and silver (Ag) nanoflakes into silicone rubber (SR) to obtain conductive composites with high sensitivity (G-SR) and high conductivity (Ag-SR), respectively. As there were no rigid metallic components in the sensor, the flexibility of the sensor could be greatly enhanced.

### 2.2. FEM Modeling

**Figure 2:**
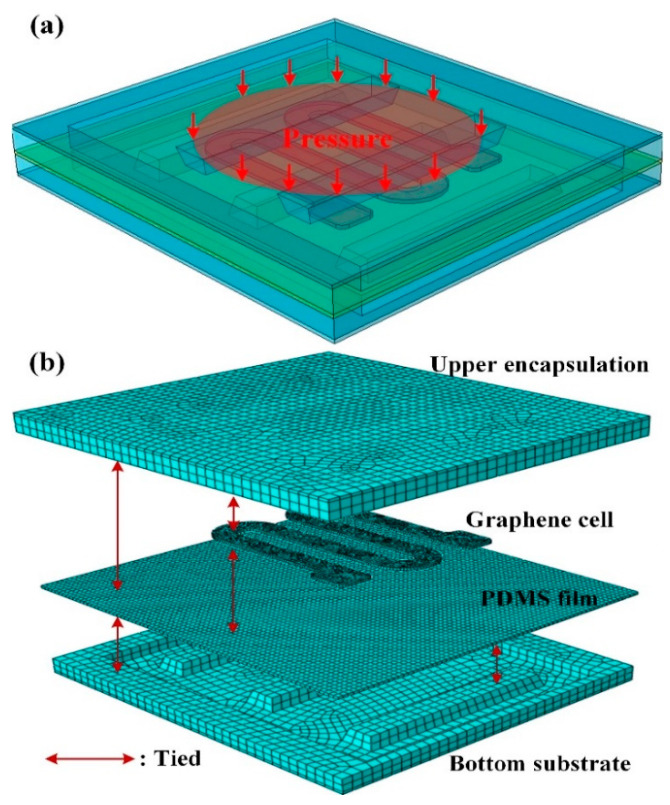
(**a**) FEM model for the tactile sensor unit; (**b**) Explosion view of the meshed unit.

To demonstrate the efficiency of the proposed tactile sensor with interlaced bumps, Finite Element Method (FEM) modeling was firstly used. The 3D model of the sensing unit (Figure 2a) was converted to the finite element mesh model via ABAQUS (v6.16, Dassault Systèmes Simulia Corp., Providence, RI, USA).

The element mesh for the multilayer structures was conducted through “structured” and “sweep” methods. The tactile sensor was divided into 41,123 hexahedron elements, and the numbers in each layer in the sensor unit were as follows: upper encapsulation (7739), graphene cell (14,370), PDMS film (11,000), and bottom substrate (8014), as shown in Figure 2b. In the FEM model, the stretchable electrode was omitted as it did not have much influence on the pressure sensing procedure. For boundary conditions, the lower surface of the bottom substrate was fixed. The contact regions among the multiple layers inside the unit were set to “tie” constraint to lock the nodes onto the contacting surfaces. A uniform pressure load of 200 kPa was applied to the top surface of the upper encapsulation, and the loading area was a circular surface with a diameter of 5 mm.

**Figure 3:**
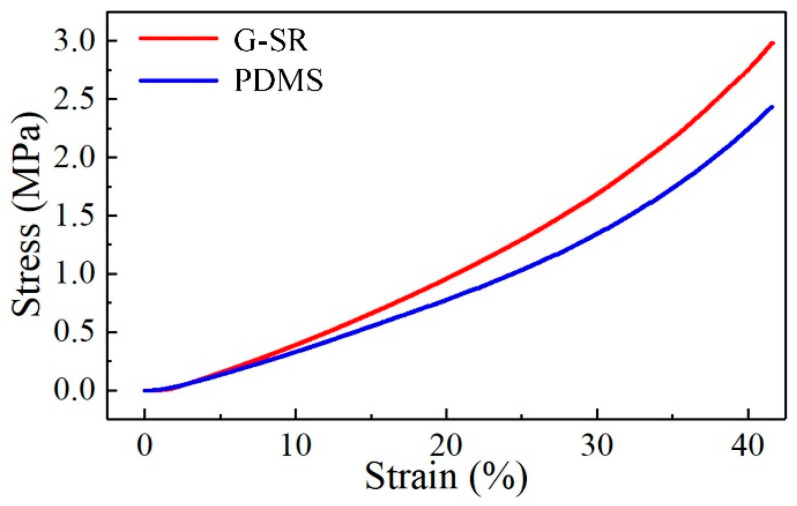
Measured stress versus strain curves of the PDMS and G-SR composites.

To obtain the mechanical properties of the PDMS and G-SR composites, a uniaxial compression test was implemented. The measured relationship between the stress and strain was provided in Figure 3. As shown in the figure, both the PDMS and G-SR composites exhibited nonlinear elastic behaviors, so the hyper-elastic Mooney-Rivlin model was selected in the ABAQUS software and the acquired curves in Figure 3 from the uniaxial compression test was uploaded into the model to represent the nonlinear properties of the materials.

## 3. Experimental Setup and Procedure

### 3.1. Composite Preparation

To prepare the G-SR and Ag-SR composites, the GNP and Ag were purchased from The Sixth Element Materials Technology Co., Ltd. (Changzhou, China), and Nanjing XFNANO Materials Tech Co., Ltd. (Nanjing, China), respectively. The silicone rubber (SR, GD401) utilized as the polymeric matrix of the composites was provided by the Zhonghao Chenguang Research Institute of Chemical Industry (Zigong, China). It is a single-component rubber that can be cured under heating and no other curing agents need to be added. The GNP with a mass fraction of 5% was added into 0.5 g SR, and 0.5 g tetrahydrofuran (THF, provided by Sinopharm Chemical Reagent Co., Ltd., Shanghai, China) was then added to enhance the dispersion uniformity of GNP and adjust the viscosity of the mixture. The above mixture was blended in a planetary mixer (AR100, Thinky Corporation, Tokyo, Japan) for 3 min, so a homogeneous and pasty uncured G-SR composite was acquired. To characterize the properties of the composites, specimens with dimensions of 20 mm × 1 mm × 0.2 mm were firstly fabricated through a screen-printing process. A glass wafer covered by a polyimide (PI) film was prepared and a 100 μm layer of PDMS (Sylgard 184, Dow Corning Co., Ltd., Midland, MI, USA, the mixing ratio of the base resin and curing agent is 10:1) was coated with a film applicator. The PDMS film was cured at 80 °C for two hours, and a steel mask with a hollow of 20 mm × 1 mm was covered on the PDMS film. The aforementioned uncured G-SR composites were coated onto the steel mask with a scraper blade. After the removal of the mask, the G-SR composites shaped in a rectangle of 20 mm × 1 mm was left and then cured under 80 °C for one hour. Finally, the cured G-SR composites were cut and peel off together with the PDMS film. As the steel mask had a thickness of 100 μm, so the composites possessed the same thickness, and the fabricated G-SR specimen acquired a total thickness of 200 μm. As for the Ag-SR composites and specimens, the same method was utilized to uniformly disperse Ag into SR with a mass ratio of Ag: SR = 2.5:1.

### 3.2. Tactile Sensor Fabrication

**Figure 4:**
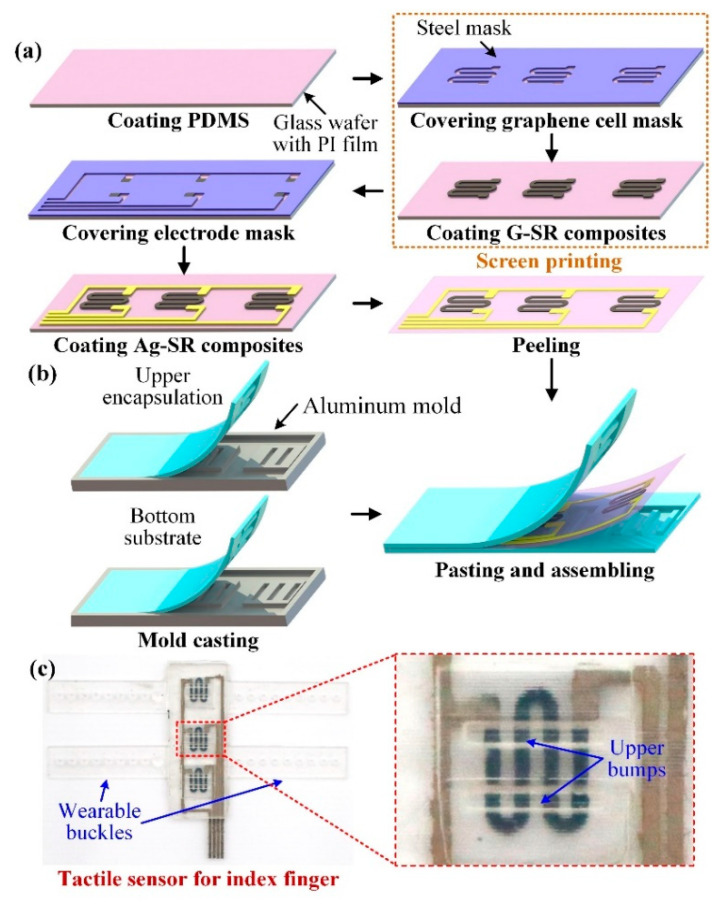
The fabrication process of the tactile sensor. (**a**) Screen-printing method for the graphene cell and stretchable electrodes; (**b**) Mold-casting of the upper and bottom layers and the assembly of the entire tactile sensor; (**c**) Images of the fabricated tactile sensor.

The fabrication process of the tactile sensor is illustrated in Figure 4. The fabrication can be included in the following four steps.

Step 1: As shown in Figure 4a, the glass wafer with PI film was coated with a 100 μm layer of PDMS film with a film applicator, and a steel mask with graphene cell patterned hollows was covered onto the film.

Step 2: The prepared G-SR composites were coated onto the mask. After removal of the mask, the graphene cells on the PDMS film were cured under 80 °C for one hour.

Step 3: The stretchable electrodes were fabricated through the same method to screen-print the Ag-SR composites onto the film. Then the PDMS film with graphene cells and stretchable electrodes were peeled off from the glass wafer.

Step 4: The designed upper encapsulation and bottom substrate with the interlaced bumps were fabricated by a typical mold-casting procedure. The aluminum molds with patterned grooves were firstly customized and the uncured PDMS was poured into the molds. After being degassed in a vacuum chamber, the molds were heated at 80 °C for two hours to cure the PDMS. Then the upper encapsulation and bottom substrate were peeled off from the molds, as shown in Figure 4b. To assemble the tactile sensor, the peripheral edges of the upper and bottom layers were coated with a thin layer of uncured PDMS. Then the upper and bottom layers were pasted to the PDMS film with graphene cells and stretchable electrodes fabricated in Step 3 after position alignment, and the sensor was further cured for another two hours to be fully assembled. Due to the identical material of the upper and bottom layers as well as the middle film, the PDMS thin layer was able to generate strong and reliable interfacial bonding between the layers, so the reliability of the assembled tactile sensor could be promoted.

The fabricated tactile sensor for the index finger has been shown in Figure 4c. From the close-up view of the sensing unit in the middle, as indicated by the red dashed rectangle, the black graphene cell has been properly connected with the gray electrodes, and the bumps on the upper encapsulation can be also observed, demonstrating a good fabrication quality of the tactile sensor.

### 3.3. Characterization of Flexible Tactile Pressure Sensor

Before the study of the sensing performance of the tactile sensor, it is necessary to characterize the electromechanical properties of the prepared G-SR and Ag-SR composites. The tensile test has been conducted to the composite specimens fabricated in Section 3.1 on a uniaxial universal testing machine (UTM 2203, Shenzhen Suns Technology Stock Co. Ltd., Shenzhen, China). Conductive tapes were linked to the two ends of the specimens with silver paste to function as peripheral electrodes, and a digital multimeter (34465A, Keysight, CA, USA) was utilized to measure the resistance of the specimens.

**Figure 5:**
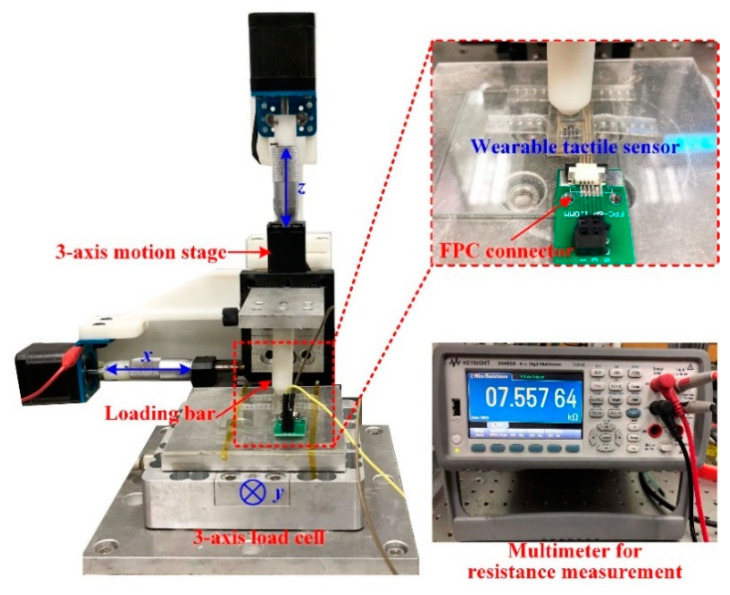
The performance testing platform setup for the tactile sensor.

To investigate the pressure sensing performance of the tactile sensor, a testing platform has been established. As shown in Figure 5, the tactile sensor was mounted on a 3-axis load cell (3A120, Interface, AZ, USA) with a resolution of 0.01 N and measurement range of 0–50 N. An aluminum loading bar (the diameter of the contacting area with the sensor is 5 mm) was fixed on the 3-axis motion stage to apply incremental pressures to the sensor. The tactile sensor was connected with an FPC connector for resistance measurement with the multimeter. The loading bar was controlled by the motion stage to implement linearly varying displacement loading on the sensing unit for the characterization of the tactile sensor. The pressure applied to the sensor was measured by the load cell.

To intuitively study the deformation of the tactile sensor during the pressure sensing unit, the sensing unit was cut apart from the center in the direction perpendicular to the bumps. The cross-sectional surface was observed by a laser confocal microscope (OLS 4100, Olympus, Tokyo, Japan) with a magnification of ×5.

### 3.4. Robotic Hand Grasping Experimental Setup and Procedure

**Figure 6:**
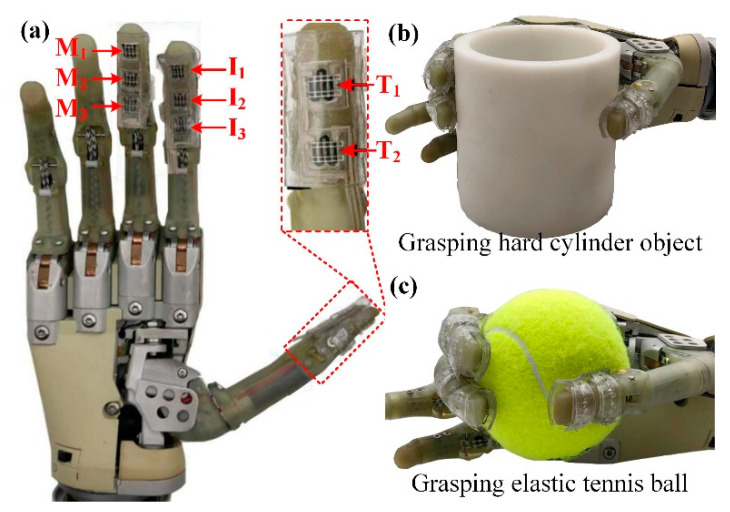
(**a**) Image of the tactile sensor worn on the humanoid robotic hand; (**b**) Grasping a hard cylinder object; (**c**) Grasping an elastic tennis ball.

The fabricated tactile sensors were attached to the fingers of the robotic hand using the buckles, as shown in Figure 6. The utilized robotic hand is an underactuated hand, which is controlled by two electromyographic signal acquisition electrodes to change its grasping states. Users can touch the electrodes to actuate the robotic hand to achieve the closing and opening motions of the fingers. The sensing units on the thumb were labeled as *T*_1_ and *T*_2_, the units on the index and middle finger were named as *I*_1_, *I*_2_, *I*_3_, *M*_1_, *M*_2_, *M*_3_, respectively from the fingertip to the finger pulp. The tactile sensor on the fingers was connected to three FPC connectors and further linked to a multichannel resistance scanner (AT 5130, Changzhou Applent Instruments, Changzhou, China) to record the resistances of all the units in the sensor. As shown in Figure 6b,c, the robotic hand integrated with the tactile sensor was controlled to grasp a 3d-printed hard cylinder object and an elastic tennis ball, respectively. In the grasping process, the robotic hand gradually grasped the objects for 2 s, steadily held it for 3 s, and then released them. The resistances of the units in the tactile sensor were measured by the multichannel resistance scanner.

## 4. Results and Discussion

### 4.1. FEM Simulation Results

**Figure 7:**
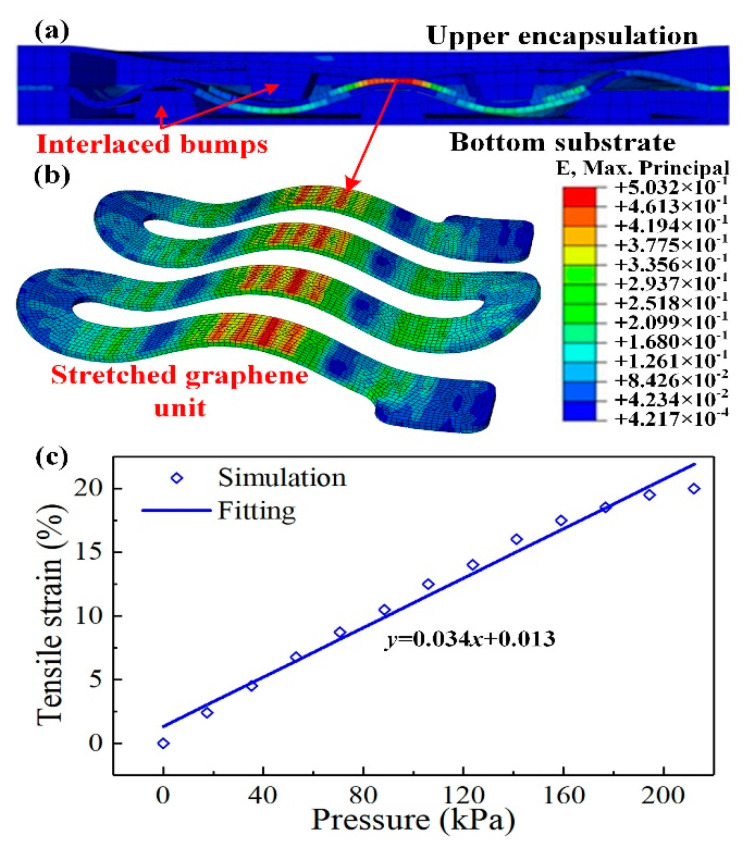
The FEM simulation results of the tactile sensor. (**a**) The cross-sectional view of the FEM model; (**b**) Overall view of the graphene cell when the outer pressure was applied; (**c**) Relationship between the tensile strain of the graphene cell versus the applied pressure.

Through the developed FEM simulation model, the pressure sensing performance of the tactile sensor can be preliminarily investigated, and the simulation results are given in Figure 7. The cross-sectional view of the sensing unit is shown in Figure 7a to expressly illustrate the deformation behavior of the unit when the outer pressure was applied. The interlaced bumps in the upper and bottom substrate squeezed the graphene cell together with the PDMS film into the gaps between the bumps and stretched the graphene cell, which is consistent with the assumption in Figure 1d. The overall view of the graphene cell is given in Figure 7b and the strain nephogram can clearly describe the deformation of the cell. As can be seen in the figure, the graphene cell was deformed to a wave-like form, and the strains were mainly distributed in the contacting areas with the interlaced bumps. The largest strain appeared on the contacting area with the middle bump in the bottom substrate, which might reach about 50% strain. Thus, the resistance of the graphene cell would dramatically increase. Through the strain nephogram, the efficiency of the proposed interlaced bumps to convert outer pressure into the stretching of the graphene cell can be easily verified. To quantitatively study the sensing performance of the tactile sensor in the FEM model, incremental pressures were gradually applied to the top surface of the unit, and the average tensile strain of the graphene cell was extracted. The relationship between the tensile strain and the applied pressure was provided in Figure 7c. As the applied pressure gradually enlarged, the tensile strain of the graphene cell monotonically increased. As shown in Figure 7c, the relationship between the pressure and the tensile strain can be fitted with good linearity with a correlation coefficient of *R*^2^ = 0.982.

### 4.2. Characterization Results of Flexible Tactile Pressure Sensor

**Figure 8:**
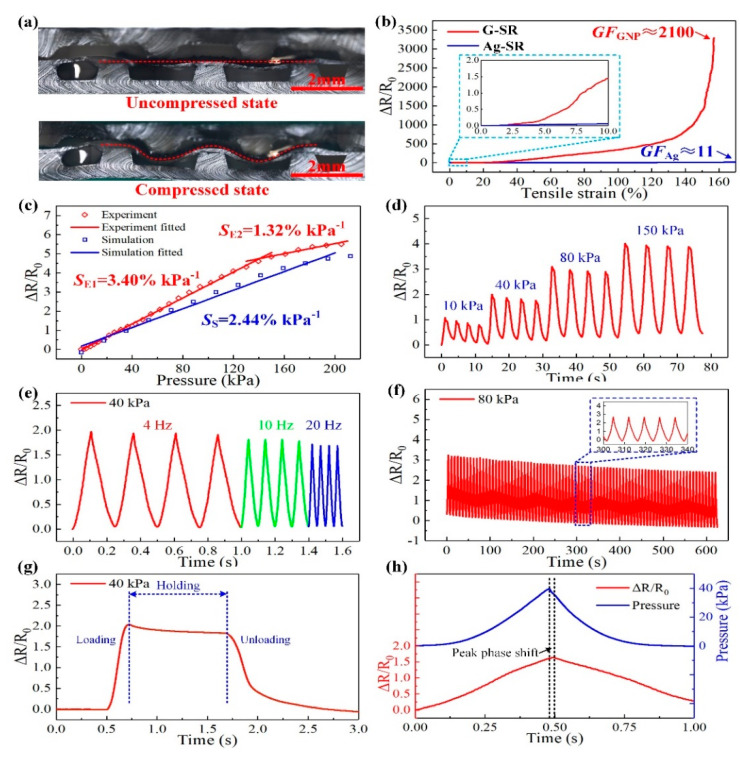
Characterization of the tactile sensor. (**a**) Optical images of the cross-section of the sensor under a laser confocal microscope; (**b**) The electromechanical properties of the G-SR and Ag-SR composites; (**c**) Calibration results of the tactile sensor; (**d**–**f**) Cyclic tests with different loading pressures, different frequencies, and multiple cycles, respectively; (**g**) Response of the unit under a steady pressure load; (**h**) Peak phase shift of the unit.

The cross-sectional images of the tactile sensor taken by the laser confocal microscope were shown in Figure 8a. The upper image is the cross-sectional view of the sensing unit without compression. The graphene cell on the PDMS film sandwiched by the interlaced bumps can be seen in the image, the upper two bumps located exactly above the gaps between the lower three bumps. The lower image describes the deformation of the sensing unit under the compressed state. The upper two bumps squeezed the graphene cell into the gaps and stretched it, which is identical to the simulation results in Figure 7a.

The electromechanical properties of the prepared G-SR and Ag-SR composites determined the sensing performance of the tactile sensor. The properties obtained from the uniaxial tensile test are given in Figure 8b. Before being stretched, the G-SR specimen possessed an initial conductivity of 3.12 × 10^−2^ S/cm, while the conductivity of the Ag-SR specimen was about 200 S/cm. The much higher conductivity of the Ag-SR composites made it possible to serve as the electrodes for electrical transmission. The G-SR specimen could be stretched to a maximum elongation of 160%, while the Ag-SR specimen could be stretched to nearly 200%. The relative changes in resistance (Δ*R*/*R*_0_, Δ*R* is the resistance change and *R*_0_ is the initial resistance of the specimens, *R*_0_ for the G-SR and Ag-SR specimens are 64.1 kΩ and 10 Ω) of the G-SR and Ag-SR specimens during stretching are plotted in Figure 8b. It is obvious that the Δ*R*/*R*_0_ of the G-SR specimen monotonically rose with the increasing tensile strains, whereas, the Δ*R*/*R*_0_ of the Ag-SR specimen seemed to have almost no change. In an enlarged view indicated by the blue rectangle in Figure 8b, the Δ*R*/*R*_0_ of the Ag-SR specimen exhibited an extremely small rise when the tensile strain reached 10%, far smaller than the G-SR specimen. To assess the sensitivity of such composites under tensile strains, the gauge factor (GF) is usually utilized, which can be calculated as *GF* = (Δ*R*/*R*_0_)/*ε*, where *ε* is the strain of the composites. Thus, as shown in Figure 8b, the maximum *GF* of the G-SR specimen was calculated as *GF*_GNP_ ≈ 2100 at *ε* = 160%, and the maximum for the Ag-SR specimen was only *GF*_Ag_ ≈ 11 at *ε* = 170%. According to the simulation results in Figure 7c, the G-SR composites would be stretched to a maximum strain of about 20% during the pressure sensing process. Under such a smaller strain, the corresponding *GF*_GNP_ was about 31, and *GF*_Ag_ was only 0.78. The huge difference in *GF* demonstrated that the G-SR specimen was far more sensitive than the Ag-SR specimen. Besides, as the Ag-SR specimen possessed a high initial conductivity, several orders of magnitude larger than the G-SR specimen, the tiny GF indicated that the Ag-SR composites would remain highly conductive when withstanding large tensile strains. So it is feasible to select the G-SR and Ag-SR composites to work as the sensitive element and stretchable electrodes, respectively. And as the other parts of the sensor were all made of flexible PDMS, the flexibility of the proposed tactile sensor could be enhanced.

After the characterization of the prepared composites, the pressure sensing performance of the tactile sensor was further investigated and the results are provided in Figure 8c–h. Firstly, the calibration test for the tactile sensor was conducted to illustrate the response of the sensor to the applied external pressure. As shown in Figure 8c, as the applied pressure became larger, the Δ*R*/*R*_0_ of the sensing unit gradually increased (*R*_0_ of the unit is about 45 kΩ). When the pressure rose to 200 kPa, the rate of increase in Δ*R*/*R*_0_ got lower, indicating the unit reached a saturation region. But the Δ*R*/*R*_0_ response of the unit still behaved with good linearity in two ranges of 0–150 kPa and 150–200 kPa. The pressure sensitivity of the unit, calculated as *S* = (Δ*R*/*R*_0_)/*p* (*p* is the applied pressure), can be acquired as *S*_E1_ = 3.40% kPa^−1^ (0–150 kPa), and *S*_E2_ = 1.32% kPa^−1^ (150–200 kPa) according to the experimental data. The *R*^2^ for the two linear fitting results is 99.8% and 97.6%, respectively. In the meanwhile, the theoretical sensitivity from the FEM model in Figure 2 can be also calculated by substituting the Δ*R*/*R*_0_ versus strain curve (Figure 8b) into the strain versus pressure curve (Figure 7c) from the FEM model. Thus, the theoretical sensitivity of the tactile sensor was obtained as *S*_S_ = 2.44% kPa^−1^ according to the FEM simulation results. The deviation between the simulation and experimental results can be mainly induced by the errors in the fabrication process of the sensor. However, through the optical images of the cross-section of the unit and the calibration results, the FEM model is generally effective to describe the sensing behavior of the tactile sensor.

Moreover, repeated cyclic tests have been implemented to the sensor to verify its good repeatability. As shown in Figure 8d, a multiple cycle test with incremental pressure loading was conducted. Pressures of 10 kPa, 40 kPa, 80 kPa, and 150 kPa were respectively applied to the sensor for 4 times. The Δ*R*/*R*_0_ of the unit changed regularly and behaved with the same varying tendency with the applied pressures. The values of Δ*R*/*R*_0_ under different pressures were approximately consistent with the calibration result. And it can be noted that there was a small rise in the initial values under different pressures, and a slight drop in the peak values under the same pressure, which can be attributed to the viscoelasticity and hysteresis of the G-SR composites. A similar phenomenon has been reported in some related studies [37,38]. Then the cyclic test with incremental loading frequencies was conducted. As shown in Figure 8e, the frequency of the 40 kPa pressure gradually increased from 4 Hz to 20 Hz, and the corresponding Δ*R*/*R*_0_ of the unit closely followed this variation and exhibited faster response. This behavior can demonstrate that the tactile sensor possessed a good dynamic response. Then a multiple cycle test of 80 cycles was implemented under 80 kPa and the output of the unit was plotted in Figure 8f. The unit showed regularly varied Δ*R*/*R*_0_, although the initial and peak values of the Δ*R*/*R*_0_ gradually declined, which could be mainly induced by the hysteresis of the G-SR composites. In the adjacent two cycles, the average drops of the initial and peak values were about 1.8% and 3.2%, respectively, and the relative change of Δ*R*/*R*_0_ remained approximately consistent during all the cycles. A sustained pressure of 40 kPa was applied to the unit, as shown in Figure 8g, the response times of loading and unloading were about 150 and 350 ms, respectively. The pressure was sustained for about 1 s, and the Δ*R*/*R*_0_ of the unit slightly declined to a steady state with a decrease of about 8%. This could be mainly due to the creep effect of the composites. Finally, the peak phase shift characteristic of the tactile sensor was shown in Figure 8h. A triangular-shaped displacement loading of 1 s was implemented to the unit, the corresponding pressure loading and Δ*R*/*R*_0_ of the unit were plotted. The pressure and Δ*R*/*R*_0_ were approximately in a triangular shape, and when the loading frequencies got higher, the triangular-shaped signals were more obvious, as shown in Figure 8e. As indicated by the black dashed lines in Figure 8h, the peak value of the pressure loading was about 0.02 s earlier than the peak of the Δ*R*/*R*_0_, demonstrating a relatively low peak phase shift of 2%, which can be negligible.

A comparison of the pressure sensitivity and sensing range of the tactile sensor in our work and those in some other related research has been conducted in Table 1, demonstrating that the proposed tactile sensor in this paper performed with relatively high sensitivity (3.40% kPa^−1^) in a large range. In overall consideration, the fabricated tactile sensor performed with relatively high sensitivity, wide sensing range, good dynamic response, and considerable repeatability. Thus, the tactile sensor will have good potential in tactile perception during object grasping of the robotic hand.

### 4.3. Robotic Hand Grasping Experimental Results

**Figure 9:**
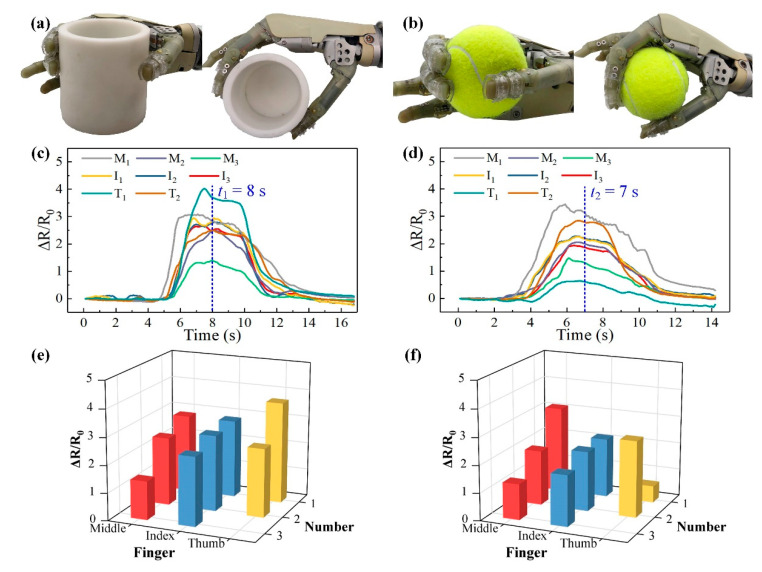
Object grasping experimental results for the tactile sensor. (**a**,**b**) Photographs of the robotic hand grasping the cylinder and tennis ball, respectively; (**c**,**d**) The Δ*R*/*R*_0_ curves during the whole grasping process for the two objects; (**e**,**f**) The distributed tactile information at the holding stage for the two objects.

To carry out the object grasping experimental studies on the humanoid robotic hand, the fabricated tactile sensor was worn onto the fingers with the help of the wearable buckles, as shown in Figure 6. Two objects have been utilized in this section: a 3d-printed hard cylinder, and an elastic tennis ball. The grasping procedure mainly included the following steps: grasping, holding, and releasing. The holding stage meant that the robotic hand steadily grasped the object and maintained for several seconds. The steady grasping state in the holding stage for the selected two objects is shown in Figure 9a,b. The Δ*R*/*R*_0_ of each unit in the tactile sensor measured by the multichannel resistance scanner in the whole grasping process for the two objects is plotted in Figure 9c,d. For the grasping process of the cylinder, as shown in Figure 9c, the robotic hand gradually grasped the cylinder from about 5–7 s, then holding for 3 s, and released from 10 s. In the grasping stage, it can be noted that the Δ*R*/*R*_0_ of the unit *M*_1_ firstly increased, indicating that the tip of the middle finger got in contact with the cylinder for the earliest. In the holding stage, the Δ*R*/*R*_0_ of all the units got their maximum values and gradually declined, induced by the hysteresis of the composites. At the steady grasping time of *t*_1_ = 8 s, the distributed Δ*R*/*R*_0_ responses of the tactile sensor were plotted in Figure 9e. From the figure, we can see the pressure distribution at the moment. For the tactile sensor, the unit *T*_1_ on the thumb fingertip exhibited the highest Δ*R*/*R*_0_, indicating that the thumb fingertip contributed the largest grasping force to the cylinder. On the contrary, the unit *M*_3_ had the lowest Δ*R*/*R*_0_, so we can infer that the finger pulp corresponding to *M*_3_ was partially in contact with the cylinder under this grasping motion. As for the other units, they showed the similar Δ*R*/*R*_0_ and illustrated that the corresponding areas on the robotic hand were uniformly withstanding the grasping forces. In the releasing stage from 10–12 s, the Δ*R*/*R*_0_ of all the units identically decreased to their original states.

When the robotic hand grasped the elastic tennis ball, as shown in Figure 9b,d, the grasping stage lasted from about 2–6 s, double longer than the stage during cylinder grasping. This could be caused by the elasticity and softness of the tennis ball, as the tennis ball would partially be deformed when being grasped by the robotic hand, so a longer time was needed to reach the steady grasping state. Besides, in this grasping stage, the unit *M*_1_ still got in contact with the ball for the earliest and the unit *T*_1_ was the latest. Through this observation, we can speculate that during the grasping process, the middle fingertip firstly touched the tennis ball and pushed the ball towards the palm to grab it. In this motion, the other parts of the robotic hand fingers gradually touched the ball and provided sufficient grasping forces. Moreover, as the thumb of the robotic hand was not able to bend, so the thumb fingertip was the last part to touch the ball. From about 6–8 s, the robotic hand got into the holding stage and the tennis ball was steadily grasped. At the moment of *t*_2_ = 7 s, the distributed tactile information was shown in Figure 9f. It is clear that during this stage, the finger pulp of the middle and thumbs, corresponding to the units *M*_1_ and *T*_2_, contributed the largest grasping forces. In the meanwhile, the thumb fingertip provided the smallest force as it got only partially in contact with the tennis ball, which can be observed in the grasping images in Figure 9b. Besides, comparing to the curves measured during hard cylinder grasping, the declining effect of the Δ*R*/*R*_0_ from the units were more obvious. This is because the tennis ball is soft and elastic, and the stress relaxation phenomenon of such objects is far more severe than the hard objects. This indicates that the grasping forces during the holding stage would slowly decrease, and result in the continuous decline of the Δ*R*/*R*_0_ from the units. As for the releasing stage from about 8 s, the sequence of the units getting detached from the tennis ball was just opposite to the grasping stage.

**Figure 10:**
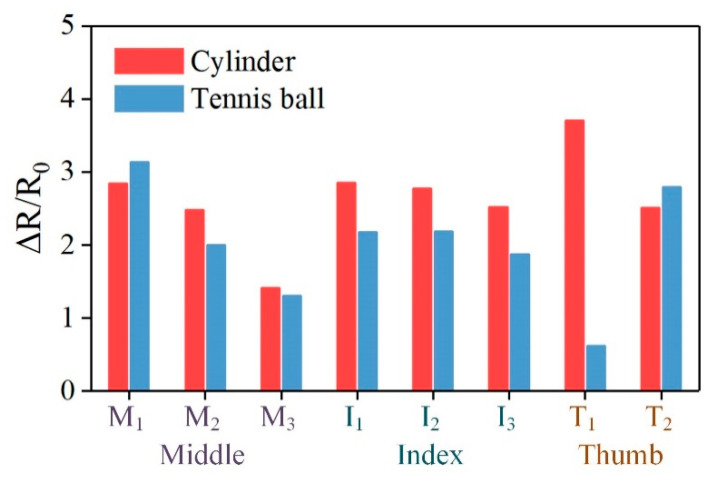
Comparison between the Δ*R*/*R*_0_ at the holding stage for the cylinder and tennis ball.

Furthermore, the horizontal comparison between the holding stages of the two objects was conducted and shown in Figure 10. The Δ*R*/*R*_0_ of all the units at the moment that the robotic hand steadily held the objects (*t*_1_ = 8 s for the cylinder and *t*_2_ = 7 s for the tennis ball) were plotted. It is obvious that the values of Δ*R*/*R*_0_ for the cylinder were mostly higher than the values of the tennis ball, so we can know that the grasping force needed for the cylinder was larger. On the other hand, during the cylinder grasping, the thumb fingertip provided the largest force while the middle finger pulp gave the smallest. As for the tennis ball grasping, the middle and thumb fingertip exhibited the largest and the smallest forces, respectively. So the difference between the grasping motions for the two objects can be inferred, as aforementioned.

From the above grasping experimental results and analysis, it is clear that the proposed tactile sensor can be well applied for tactile perception during robotic hand grasping. Through the acquired tactile information in the time and spatial domains, the grasping modes and motions of the robotic hand can be studied. Furthermore, the characteristics of the output response from the units will be beneficial to the deeper investigation of the object properties, such as hardness and softness. In general, the above experimental results have effectively proven the great potentials of this tactile sensor for intelligent robotics.

## 5. Conclusions

In summary, we proposed and developed a tactile sensor with bilayer interlaced bumps for robotic grasping applications. The entire tactile sensor was designed and fabricated based on elastic polymers. The graphene and silver nanoflakes were dispersed into the silicone rubber to serve as the pressure sensing elements and stretchable electrodes, respectively. The homogeneous G-SR and Ag-SR composites showed distinctly different electrical properties, so the stretchable electrodes would not generate much influence on the sensing elements and insured the sensing accuracy. The proposed bilayer interlaced bumps were designed to convert the external pressures into the stretching of the graphene composites, which were verified by the FEM simulation results. Besides, the interlaced bump structure also helped to balance the sensitivity and sensing range of the tactile sensor, showing the results of relatively high sensitivity of 3.40% kPa^−1^ in a wide range of 0–150 kPa, and 1.32% kPa^−1^ in 150–200 kPa. The tactile sensor has performed with good repeatability in 80 cycles with average drops of the initial and peak values of 1.8% and 3.2%. The sensor could well respond to the loadings of up to 20 Hz. The response time was about 150 ms and the peak phase shift between the pressure loading and the output of the sensor was about 2%. The tactile sensor has been successfully integrated with the humanoid robotic hand to grasp a hard cylinder object and an elastic tennis ball. The distributed tactile information during the whole grasping process could be acquired by the tactile sensor. Through the analysis of the tactile information in both time and spatial domains, the grasping motions and the properties of the grasping objects, like hardness and softness, could be investigated. Furthermore, the relations between the tactile information and the grasping modes, motions, objects can be deeply studied through the tactile sensor in this paper, which will be our future research focus.

## Figures and Tables

**Table 1:** The comparison of the sensing performance in this paper and some other related works.

Ref.	Materials	Sensing Mechanism	Sensitivity	Sensing Range
[4]	Silicon nanoribbon-PDMS	Piezoresistive	0.41% kPa^−1^	200 kPa
[9]	AgNW/Dragonskin	Piezoresistive	0.3% kPa^−1^	200 kPa
[39]	Carbon nanocoil-PDMS	Piezoresistive	0.076% kPa^−1^	100 kPa
[33]	Graphene-PAS	Piezoresistive	1.1% kPa^−1^	30 kPa
[40]	PDMS-Ni fibers	Piezoresistive	0.0053 kPa^−1^	72 kPa
[7]	GNP/CNT/SR/PU	Capacitive	6.2% kPa^−1^	4.5 kPa
[41]	AgNF-AgNW	Capacitive	0.18% kPa^−1^	1.6 MPa
[11]	AgNF/Silk	Capacitive	1.89% kPa^−1^	700 kPa
[42]	PbTiO3NW-Graphene	Piezoelectric	0.94% kPa^−1^	1.4 kPa
[43]	ITO-Graphene FET-PDMS	Triboelectric	2% kPa^−1^	57 kPa
This work	GNP-SR-PDMS	Piezoresistive	3.40% kPa^−1^ 1.32% kPa^−1^	0–150 kPa 150–200 kPa
